# tNGS-based detection of respiratory pathogens in a single center: associations with age, gender, season, and co-infections

**DOI:** 10.3389/fcimb.2025.1663234

**Published:** 2025-11-25

**Authors:** Qingling Wang, Dan Wu, Yanzi Zhang, Qian Zeng, Juan Wang, Xin Lv

**Affiliations:** 1 Clinical Laboratory, Children’s Hospital Affiliated to Shandong University, Jinan, China; 2 Clinical Laboratory, Jinan Children’s Hospital, Jinan, China

**Keywords:** targeted next-generation sequencing, respiratory pathogens, age distribution, seasonal variation, co-infection

## Abstract

**Background:**

Respiratory tract infections represent a significant global health challenge. Conventional diagnostic methods frequently fail to detect complex infections or novel pathogens. This study employed Targeted Next-Generation Sequencing to achieve an unbiased and comprehensive identification of respiratory pathogens, as well as to conduct analysis of pathogen distribution across age, gender and seasons.

**Methods:**

We conducted a retrospective analysis of clinical samples, including throat swabs, sputum, and bronchoalveolar lavage fluid, obtained from symptomatic patients. The analysis utilized targeted next-generation sequencing in conjunction with bioinformatics. Statistical assessments were performed to evaluate associations with age, gender, season, and co-infections, primarily employing Chi-square tests.

**Results:**

A high pathogen detection rate of 97.08% was achieved among 20059 individuals. Bacteria were the most frequently detected pathogens, accounting for 49.62%, followed by viruses at 43.31%, and special pathogens at 7.07%. Significant age-related differences in pathogen profiles were observed. Although no overall gender effect was detected, variations specific to certain pathogens were noted. Clear seasonal trends emerged for key pathogens. Co-infections were highly prevalent, with bacterial-viral combinations being the most common, affecting 49.03% of patients, which exceeded the rate of bacterial infections alone at 15.69%.

**Conclusion:**

Targeted next-generation sequencing serves as a robust tool for elucidating the intricate spectrum and epidemiology of respiratory pathogens. This study underscores significant associations with patient age, seasonal variations, and the prevalence of co-infections, providing essential insights for targeted clinical and public health interventions in response to respiratory tract infections.

## 1 Introduction

Respiratory tract infections are among the leading causes of morbidity and mortality worldwide. The diverse pathogens and overlapping clinical manifestations associated with these infections present significant challenges for accurate diagnosis (Tenenbaum, 2021; Li et al., 2025b; Li et al., 2025a). Traditional microbial detection methods, including culture, antigen detection, and PCR, often fall short in meeting the demands for rapid diagnosis due to limitations such as sensitivity ranging from 40% to 70%, low throughput, and the inability to identify co-infections (Ma et al., 2024; Li et al., 2025b). This is particularly concerning for immunocompromised patients and vulnerable populations, such as children, where traditional diagnostic approaches can result in delays in treatment and inappropriate use of antimicrobials (He et al., 2025; Li et al., 2025b).

In recent years, targeted next-generation sequencing (tNGS) technology has demonstrated significant application value in the detection of respiratory pathogens through genome capture and enrichment strategies. Compared to metagenomic NGS (mNGS), tNGS substantially reduces the volume of sequencing data and the associated costs (by 50% to 75%) while maintaining comparable or even higher sensitivity (ranging from 84.38% to 97.73%) and specificity (between 75.41% and 92.1%) through the use of customized probe or primer combinations (Fu and Zhou, 2024; Yin et al., 2024; Zheng et al., 2024; Gu et al., 2025). Regarding co-infection detection, the positive rate (49.11% to 89.5%) is markedly higher than that of traditional methods (2.85% to 53.2%), particularly excelling in the identification of atypical pathogens (such as Mycoplasma pneumoniae) and fastidious bacteria that are often overlooked by conventional approaches (Ma et al., 2024; Dai et al., 2025; He et al., 2025). In terms of non-invasive sampling, the tNGS detection results from throat swabs and bronchoalveolar lavage fluid (BALF) samples show consistency rates of 88% to 95.8%, with costs reduced by over 40%, thereby providing a viable solution for pathogen screening in pediatric patients (Lian et al., 2025; Li et al., 2025a). Previous studies have also confirmed that tNGS plays a crucial role in guiding drug therapy, with antibiotic regimens being adjusted in 75% of cases based on test results, significantly shortening hospital stays (Dai et al., 2025; Li et al., 2025b).

The etiology of respiratory tract infections is diverse, encompassing a wide array of pathogens, including viruses such as the Influenza virus(IFV), Respiratory Syncytial Virus (RSV), Human Rhinovirus (RV), Adenovirus (AdV), Human Metapneumovirus (hMPV), and Human Parainfluenza viruses (HPIV), as well as bacteria like Streptococcus pneumoniae and Haemophilus influenzae, along with other pathogens such as Mycoplasma pneumoniae (Lv et al., 2024; Contes and Liu, 2025; Li et al., 2025; Tang et al., 2025). Targeted next-generation sequencing (tNGS) significantly enhances detection efficiency by specifically targeting the genomes of these pathogens. Existing studies indicate that the detection rate of tNGS in respiratory tract infections can reach between 84.3% and 100% (Ma et al., 2024; Li et al., 2025b).

The primary objectives of this study were: 1) to characterize the spectrum and frequency of respiratory pathogens detected by targeted next-generation sequencing (tNGS) in a cohort of patients presenting with respiratory symptoms; 2) to analyze the statistical associations between pathogen detection and patient age and gender; 3) to describe the temporal distribution of identified pathogens on a monthly and seasonal basis; 4) to investigate the prevalence and patterns of pathogen co-infections; and 5) to discuss the epidemiological significance of these findings in understanding local respiratory tract infection (RTI) dynamics and informing public health strategies.

## 2 Materials and methods

### 2.1 Data and specimens collection

This retrospective study included 20,059 children diagnosed with respiratory tract infections, comprising 8,356 girls and 11,703 boys, aged between 1 day and 18 years. All participants were admitted to the Children’s Hospital affiliated with Shandong University from January 2022 to December 2024. The analysis incorporated pathogen detection results from targeted next-generation sequencing (tNGS) for all subjects involved in the study. Samples, including pharyngeal swabs, sputum, and bronchoalveolar lavage fluid, were collected by trained nurses in accordance with Standard Operating Procedures (SOP). These samples were promptly transported to the clinical laboratory for analysis.

### 2.2 Sample detection

#### 2.2.1 Selection of genomic targets and primer design

The target spectrum was established through a comprehensive review of multiple sources, including expert consensus documents and infectious disease literature (Huang et al., 2020; Li et al., 2021; Liu et al., 2023). Reference sequences were primarily obtained from NCBI RefSeq/NT databases and subsequently refined by eliminating highly similar redundant entries. Gene selection prioritized targets previously validated by established PCR assays, followed by bioinformatic screening to identify conserved and specific genomic regions.

Specific primers were designed according to stringent criteria adapted from a previous publication (Yin et al., 2024), which included: (1) primer length of 18–26 bp; (2) melting temperature (TM) near 60 °C; (3) GC content between 40–60%; and (4) absence of self-dimers, hairpin structures, and cross-dimers. These pathogen-specific multiplex PCR primers were developed by KingCreate Biotech (Guangzhou, China) and manufactured by Sangon Biotech (Shanghai, China). The corresponding amplification protocol was systematically optimized to achieve highly sensitive target enrichment.

#### 2.2.2 Sample processing, library preparation, and high-throughput sequencing

All clinical specimens and controls (positive, negative, and non-template controls) underwent total nucleic acid extraction using an ISO 13485-certified purification system (Nucleic Acid Extraction Kit, KingCreate Biotech, China). Purified nucleic acids were eluted in a dedicated reaction buffer (UP50 Premix Kit, KingCreate, China).

Libraries were constructed through a two-step amplification process. The initial amplification was performed under the following conditions: 95°C for 3 min; 25 cycles of 95°C for 30 s and 68°C for 1 minute. A subsequent amplification consisted of 30 cycles of 95°C for 30 s, 60°C for 30 s, and 72°C for 30 s, followed by a final extension at 72°C for 1 minute. Amplified products were purified using magnetic bead-based cleanup (UP50 Premix Kit). Quantification was carried out using the Equalbit DNA HS Assay Kit (Vazyme, China) on a Qubit™ Fluorometer 3.0/4.0 (Thermo Fisher Scientific, USA).

Sequencing libraries were prepared with an ISO 13485-certified library construction kit (General Sequencing Reaction Kit, KingCreate Biotech, China). All libraries were normalized to a minimum concentration of 0.5 ng/μL. Qualified libraries were pooled in equimolar ratios, denatured, and loaded at a volume of 500 μL onto a KM Miniseq Dx-CN Sequencer (Kingcreate, China), which is an ISO 13485-certified platform, for 2 × 150 bp paired-end sequencing. Each batch included external controls (Bacillus subtilis and saline) processed alongside clinical samples throughout all stages.

#### 2.2.3 Bioinformatic analysis

Raw sequencing data were demultiplexed and converted to FASTQ format using bcl2fastq. Quality assessment was performed using FastQC (v0.12.0) and MultiQC (v1.29) (Ewels et al., 2016) to evaluate overall sequencing performance. Adapter trimming and quality filtering were conducted with Fastp (v0.20.1) (Chen et al., 2018) under the following criteria: (1) removal of reads containing more than 10 ambiguous bases (N); (2) exclusion of reads shorter than 15 bp;(3) trimming of reads with mean quality scores below 15.

The human reference genome (hg19; GCF_000001405.13) and pathogen reference sequences were retrieved from the NCBI GenBank database. Reads were first aligned to the human genome using BWA-mem (v0.7.18) (Li and Durbin, 2009) to subtract host-derived sequences. The remaining non-human reads were then mapped against pathogen genomes using Bowtie2 (v2.4.1) in ‘very-sensitive’ mode (Langmead and Salzberg, 2012). Alignment statistics, including depth and coverage, were computed using Samtools (v1.21) (Li et al., 2009) and Bamdst (v1.0.5). To qualify for further analysis, alignments were required to exhibit over 40 bp of overlap with target amplicon regions.

Pathogen detection was based on normalized read counts. Reads mapping to each target amplicon were summed and scaled to reads per 100,000 sequenced reads (RPhK). A positive call was made for a given taxonomic unit (species or higher) if the RPhK value reached or exceeded 10; otherwise, the result was reported as “absent”.

### 2.3 Statistical analysis

The outcome variables included the detection of specific pathogens and the status of co-infection. The independent variables comprised patient age (categorized as 0-1, 1-3, 3-6, 6-10, and ≥10 years), gender (male and female), and the month or season of sample collection, which was derived from temporal data. Statistical analyses primarily utilized Chi-square (χ²) tests to compare categorical data, such as pathogen detection rates between genders and across age groups. Gender-based differences in pathogen infection rates were analyzed using Pearson’s Chi-square tests, with a significance level set at P< 0.05. Temporal trends were assessed by plotting monthly detection rates. Co-infection was defined as the simultaneous detection of two or more distinct pathogenic microbial species within the same sample. To explore seasonal variations and gender-related differences in pathogen infections, the trends in detection rates from January 2022 to December 2024 were illustrated using GraphPad Prism 8.0. Age-stratified detection patterns of the top 10 respiratory pathogens and co-detection patterns were illustrated using Origin 2024.

## 3 Results

### 3.1 Patient demographics and overall pathogen detection

Among the 19,474 pathogen-positive cases, males constituted 58.27% (11,348/19,474), compared to 60.68% (355/585) in the negative group, with no statistically significant gender difference observed (χ²=1.359, p=0.2438). Analysis of age distribution revealed significant disparities between the groups (χ²=288.2, p<0.0001). The negative group exhibited a markedly higher proportion of infants (0–1 year: 49.57% vs. 24.61%), while positive cases were more prevalent in older age categories, particularly among those aged 3–6 years (27.17% vs. 11.45%) and 6–10 years (24.15% vs. 11.80%). Notably, only 4.43% of positive cases occurred in patients aged ≥10 years, in contrast to 14.53% in the negative group. The demographic characteristics of both the positive and negative groups are summarized in **Table 1**.

**Table 1:** Comparison of demographic characteristics and specimen types.

Characteristics	Positive group	Negative group	Statistic	P
Gender	n (%)			
Male	11348 (58.27)	355 (60.68)	*χ²* = 1.359	0.2438
Female	8126 (41.73)	230 (39.32)		
Age(year)	n (%)			
0-1	4793 (24.61)	290 (49.57)	*χ²* = 288.2	<0.0001
1-3	3824 (19.64)	74 (12.65)		
3-6	5291 (27.17)	67 (11.45)		
6-10	4703 (24.15)	69 (11.80)		
≥10	863 (4.43)	85 (14.53)		
Specimen types				
Throat swabs	14720	545	*χ²* = 98.12	<0.0001
Sputum	3526	23		
Bronchoalveolar lavage fluid	1228	17		

### 3.2 Spectrum and frequency of detected pathogens

**Figure 1:**
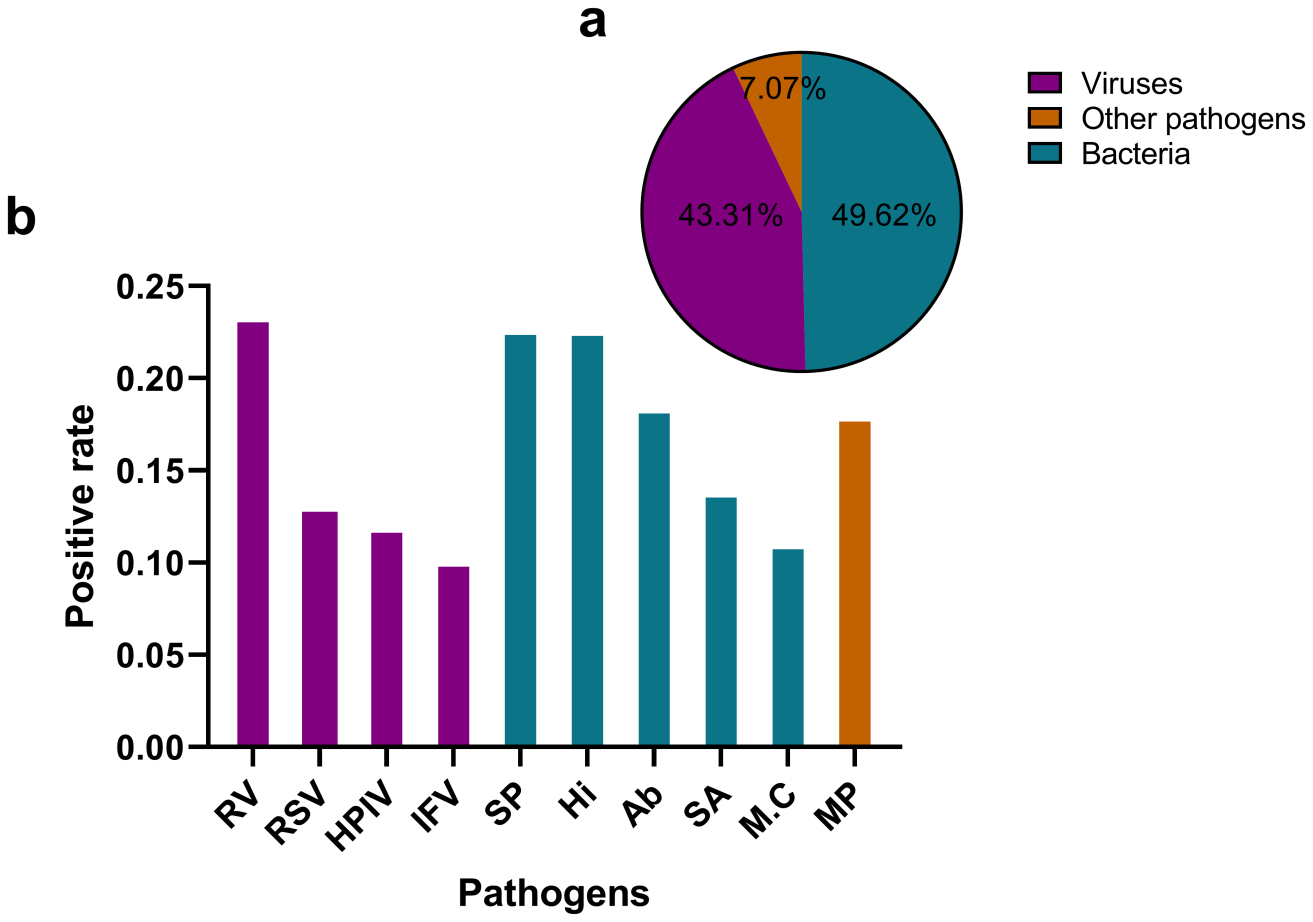
Distribution of pathogen types detected by tNGS in respiratory infections **(a)** illustrates a pie chart that delineates the distribution of various pathogen categories. Viruses constitute 43.31% of the total, while bacteria represent 49.62%. Other pathogens, which include Mycoplasma pneumoniae, Chlamydia pneumoniae, Chlamydia psittaci, and Chlamydia trachomatis, Ureaplasma urealyticum, Ureaplasma parvum, Mycoplasma hominis, Mycoplasma genitalium account for 7.07%. **(b)** presents a bar graph that depicts the positive rates of the ten most prevalent pathogens. RV, Rhinovirus; SP, Streptococcus pneumoniae; Hi, Haemophilus influenzae; Ab, Acinetobacter baumannii; MP, Mycoplasma pneumoniae; SA, Staphylococcus aureus; RSV, Human Respiratory Syncytial Virus; HPIV, Human Parainfluenza Virus; M.C, Moraxella catarrhalis; IFV, Influenza Virus.

The tNGS assay encompasses a panel of 107 respiratory pathogens and the list has been provided as a **Supplementary Table S1**. The analysis of pathogen distribution revealed distinct patterns among the detected microorganisms. Bacterial pathogens accounted for the majority of positive detections (49.62%), closely followed by viral pathogens (43.31%). Other pathogens, including Mycoplasma pneumoniae, Chlamydia pneumoniae, Chlamydia psittaci, Chlamydia trachomatis, Ureaplasma urealyticum, Ureaplasma parvum, Mycoplasma hominis and Mycoplasma genitalium represented a smaller proportion of cases (7.07%). This distribution demonstrates a predominance of bacterial etiologies in respiratory infections, with viruses serving as a significant secondary component. The relatively low percentage of special pathogens suggests that they play a less frequent, yet clinically relevant, role in respiratory infections within the studied population. The ten most frequently detected pathogens were Rhinovirus(RV), Streptococcus pneumoniae (SP), Haemophilus influenzae(Hi), Acinetobacter baumannii(Ab), Mycoplasma pneumoniae(MP), Staphylococcus aureus(SA), human respiratory syncytial virus(RSV), Human Parainfluenza Virus(HPIV), influenza virus(IFV), and Moraxella catarrhalis(M.C), with their positive detection rates illustrated in **Figure 1**.

### 3.3 Pathogen detection in relation to gender

**Figure 2:**
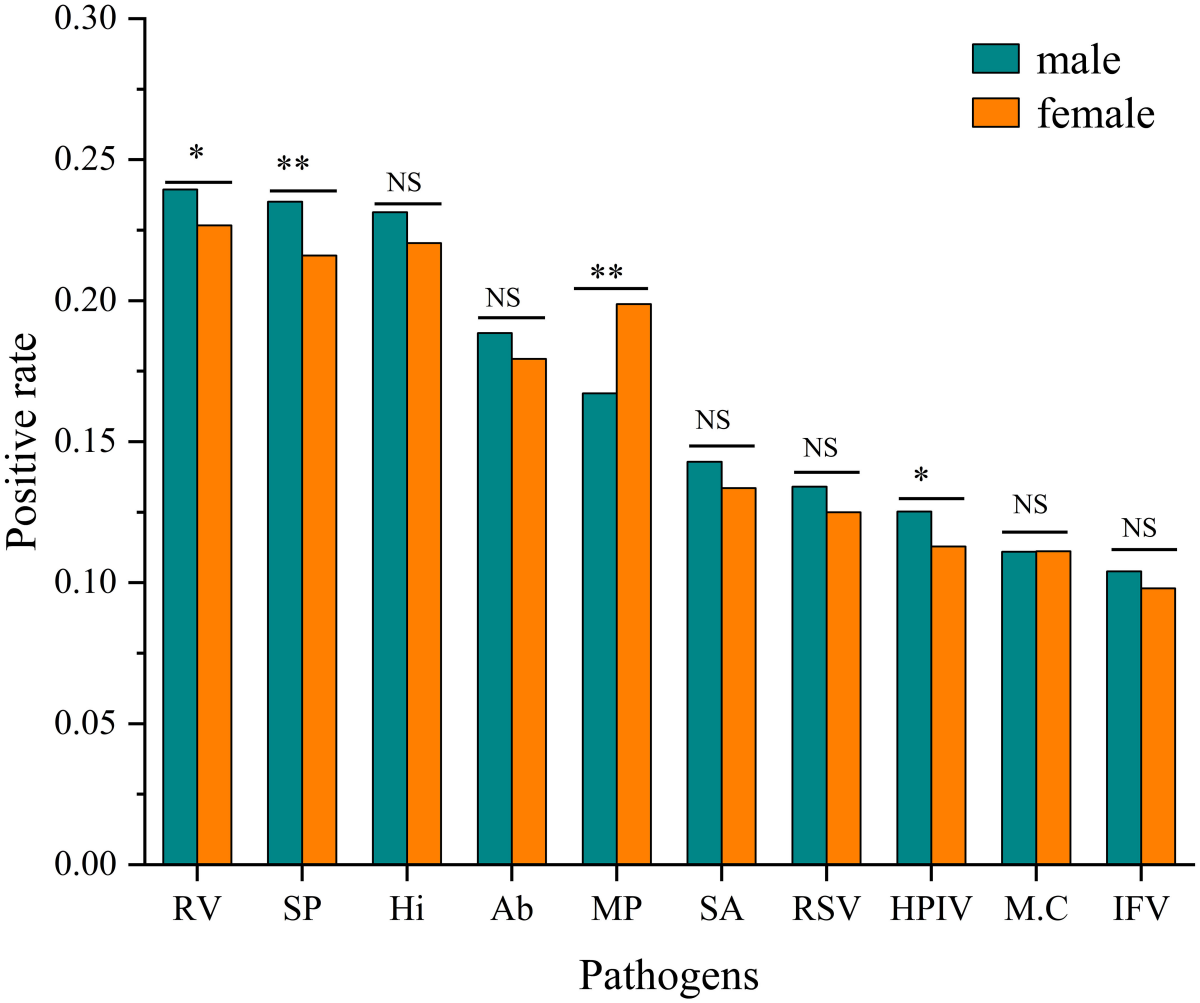
Comparison of positive rates by pathogen and gender. Infection rates for each pathogen are shown for males and females. Statistical significance of differences between genders was determined by Pearson’s Chi-square tests (*P<0.05, **P<0.001, NS, Not Significant).

Significant gender differences in positive rates were observed for four pathogens. Rhinovirus(RV) showed a higher infection rate in males (23.94% vs. 22.67%, χ² = 4.412, P< 0.05). Streptococcus pneumoniae (SP) showed a higher infection rate in males (23.51% vs. 21.60%, χ² = 10.146, P<0.001). Conversely, Mycoplasma pneumoniae (MP) demonstrated a significantly higher infection rate in females (19.88% vs. 16.72%, χ²=32.939, P < 0.001). Human parainfluenza virus (HPIV) also showed a modest but significant male predominance (12.52% vs. 11.29%, χ² = 7.049, P < 0.05). No significant gender differences were observed for the remaining six pathogens after multiple comparison adjustment. (**Figure 2**).

### 3.4 Pathogen detection in relation to age

**Figure 3:**
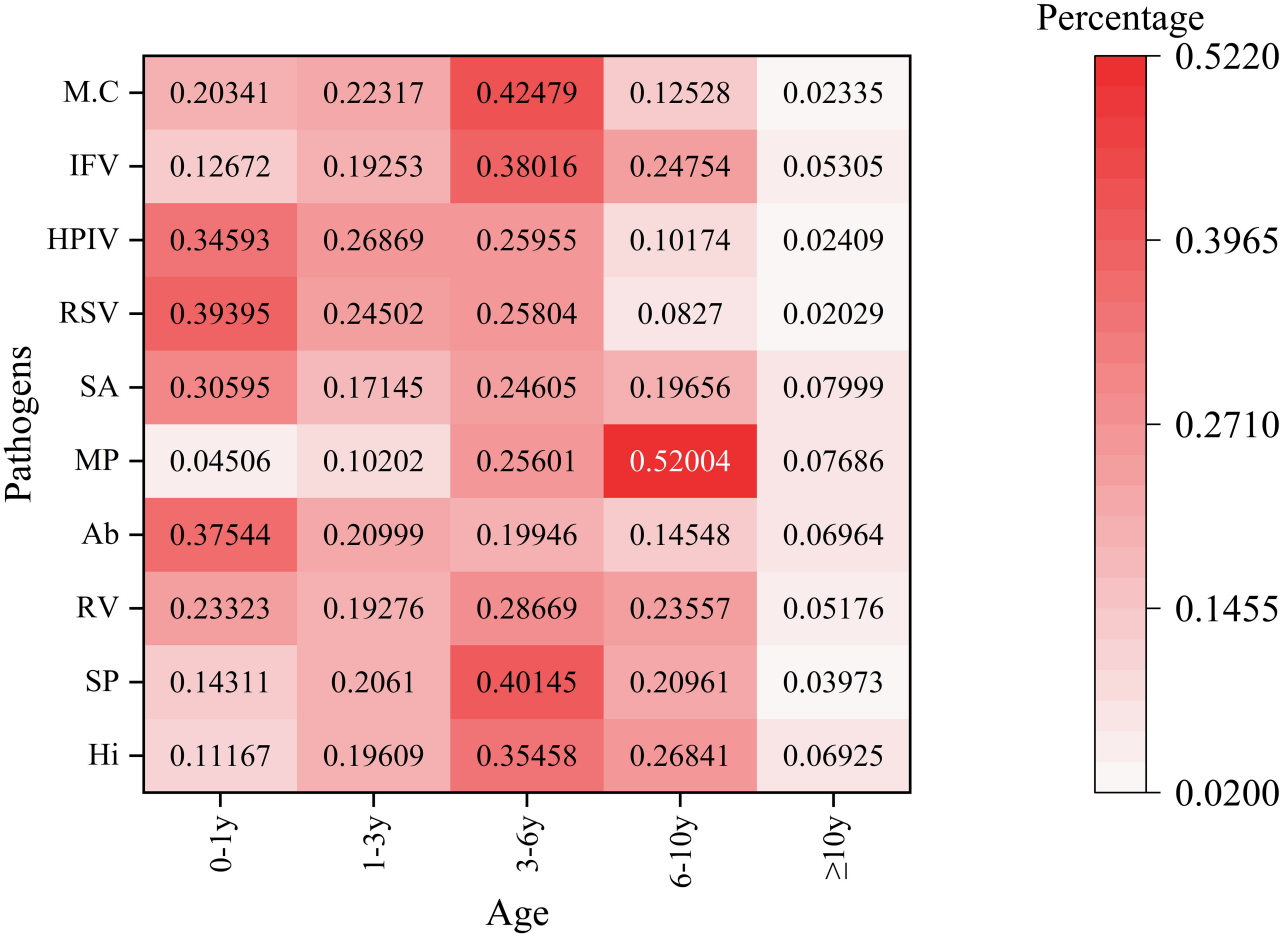
Age-stratified detection patterns of the top 10 respiratory pathogens This heatmap illustrates the percentage distribution of various pathogens across different age groups. The rows represent different pathogens. The columns categorize age groups as follows: 0 - 1y (0–1 year old), 1 - 3y (1–3 years old), 3 - 6y (3–6 years old), 6 - 10y (6–10 years old), and≥10y (10 years and older). The color intensity ranges from light pink (indicating a low percentage) to dark red (indicating a high percentage), reflecting the proportion of each pathogen within the corresponding age group. The numbers displayed on the heatmap represent the detection percentages of various pathogens across different age categories.

Pathogen detection exhibited significant variation across different age groups, as illustrated in **Figure 3**. The age categories analyzed were 0-1, 1-3, 3-6, 6-10, and ≥10 years. In the neonatal/infant group (0–1 year), human respiratory syncytial virus displayed the highest prevalence at 39.40%, followed closely by Acinetobacter baumannii at 37.54% and parainfluenza virus at 34.59%. Mycoplasma pneumoniae had the lowest detection rate in this age group, at 4.51%. Detection of Streptococcus pneumoniae peaked at 40.15% in the 3–6 year age group. Conversely, Mycoplasma pneumoniae showed an inverse trend in the 6–10 year age group, reaching a maximum detection rate of 52.00%, contrasting sharply with its low prevalence in younger cohorts. In adolescents (≥10 years), the activity of all pathogens was notably reduced.The detection rate of Haemophilus influenzae in the 3–6 year age group is relatively high. This finding may be associated with the characteristics of pharyngeal swab samples, which facilitate the detection of upper respiratory tract colonizing bacteria. Its clinical significance requires further evaluation in conjunction with the presence of infection symptoms in children.

### 3.5 Temporal distribution of pathogens

**Figure 4:**
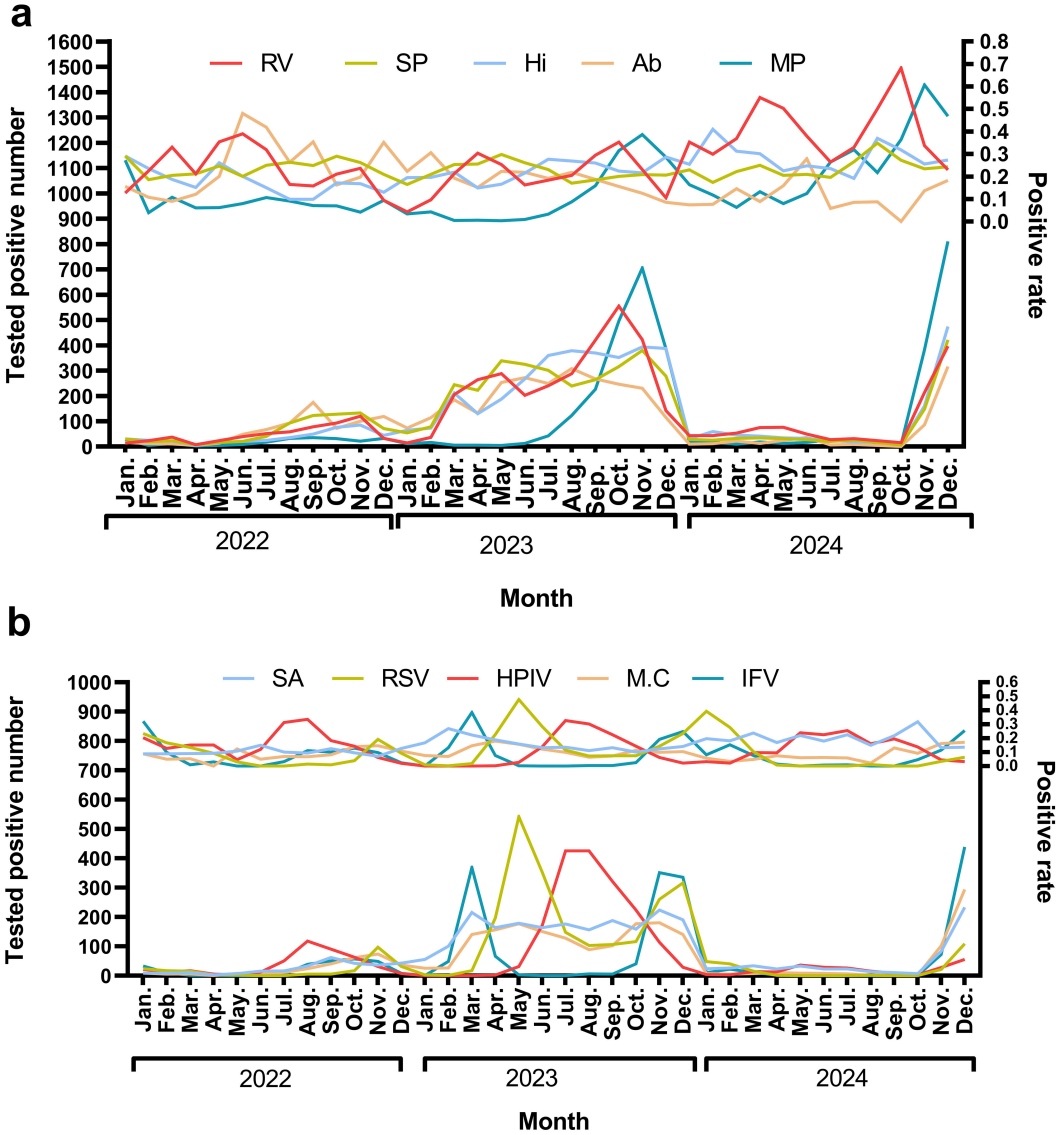
The monthly positive rate for top 10 pathogens In **(a)** the pathogens identified are Rhinovirus (RV), Streptococcus pneumoniae (SP), Haemophilus influenzae(Hi), Acinetobacter baumannii (Ab), and Mycoplasma pneumoniae(MP). The upper panel illustrates the trend in positive rates, while the lower panel depicts the trend of the number of positive tests. In **(b)** the pathogens include Staphylococcus aureus(SA), Respiratory syncytial virus(RSV), Human parainfluenza virus(HPIV), Moraxella catarrhalis(M.C), and Influenza virus(IFV). Consistent with **(a)** the upper panel shows the positive rate trends, and the lower panel indicates the number of positive tests.

Rhinovirus (RV) circulates year-round, exhibiting dual peaks during the summer (June–July) and autumn/winter (October–December). Streptococcus pneumoniae (SP) demonstrates low-level detection throughout the year, with a notable increase in September–October (27.3%–34.8%) in 2024. Haemophilus influenzae (Hi) shows high detection rates in February and September–October 2024, ranging from 36.9% to 40.9%, which are associated with seasonal outbreaks. Acinetobacter baumannii (Ab) experienced a historical peak in the summer of 2022 (48%), followed by a decline in 2024. In the autumn/winter months (October–December), there is an increase in RV, Influenza Virus (IFV), and Respiratory Syncytial Virus (RSV). IFV peaks in winter, with notable rates of 32.0% in January 2022 and 24.8% in December 2023, before declining in 2024. RSV also shows high detection rates, reaching 39.34% in January 2024. Mycoplasma pneumoniae (MP) experiences outbreaks in late autumn and winter, peaking at 38.6% in November 2023 (**Figure 4**).

### 3.6 Co- detection patterns analysis

**Figure 5:**
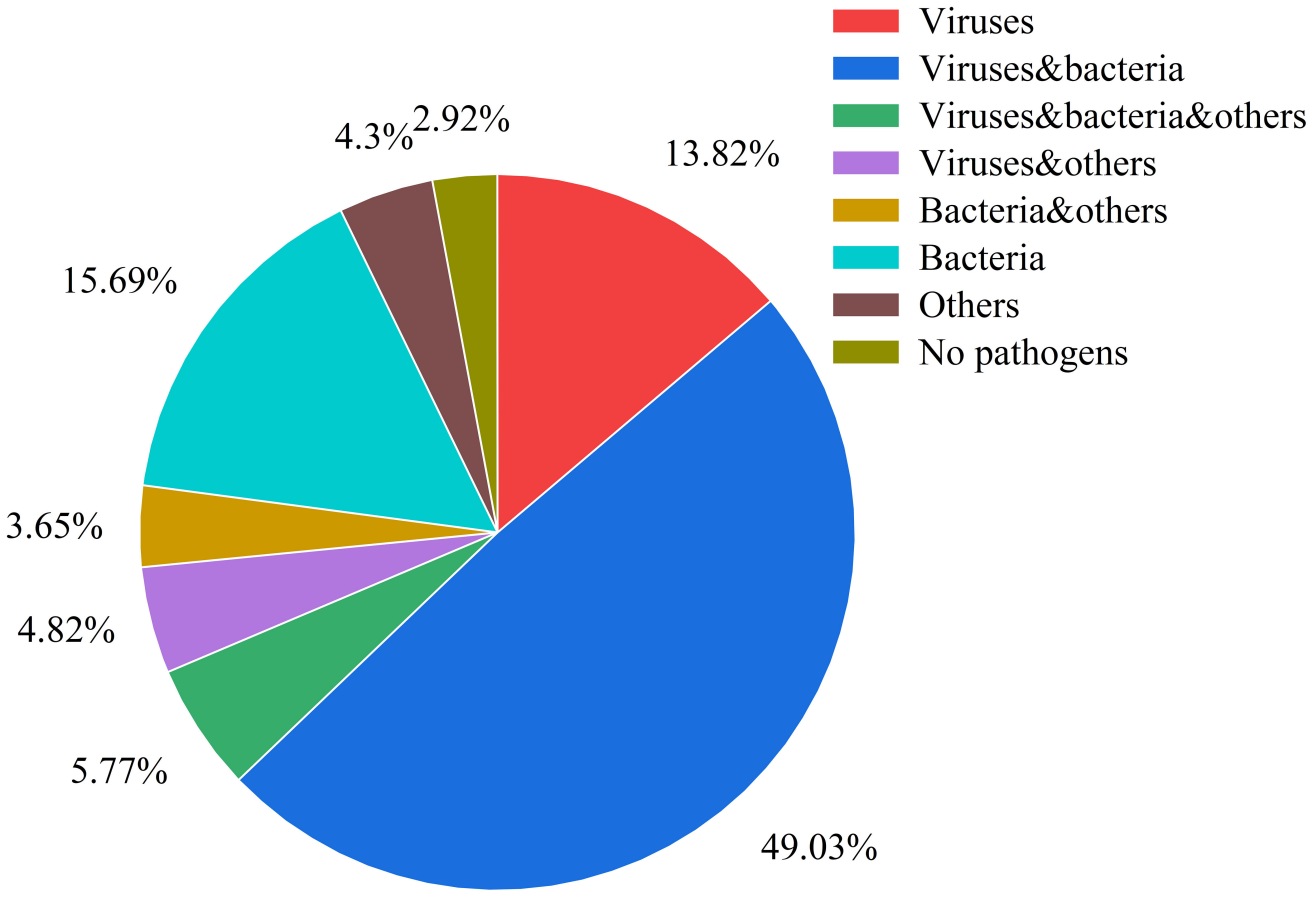
Pathogen co-detection patterns in respiratory infections This pie chart illustrates the distribution of various pathogen combinations and other categories within the samples. The categories are defined as follows: Viruses: only viruses were detected. Viruses & Bacteria: both viruses and bacteria were detected. Viruses, Bacteria & Others: detection involving viruses, bacteria, and other pathogens (including Mycoplasma pneumoniae, Chlamydia pneumoniae, Chlamydia psittaci, Chlamydia trachomatis, Ureaplasma urealyticum, Ureaplasma parvum, Mycoplasma hominis, Mycoplasma genitalium). Viruses & Others: detection involving viruses and other pathogens. Bacteria & Others: detection involving bacteria and other pathogens. Bacteria: only bacteria were detected. Others:only other pathogens were detected. No Pathogens: samples in which no pathogens were detected.

**Figure 6:**
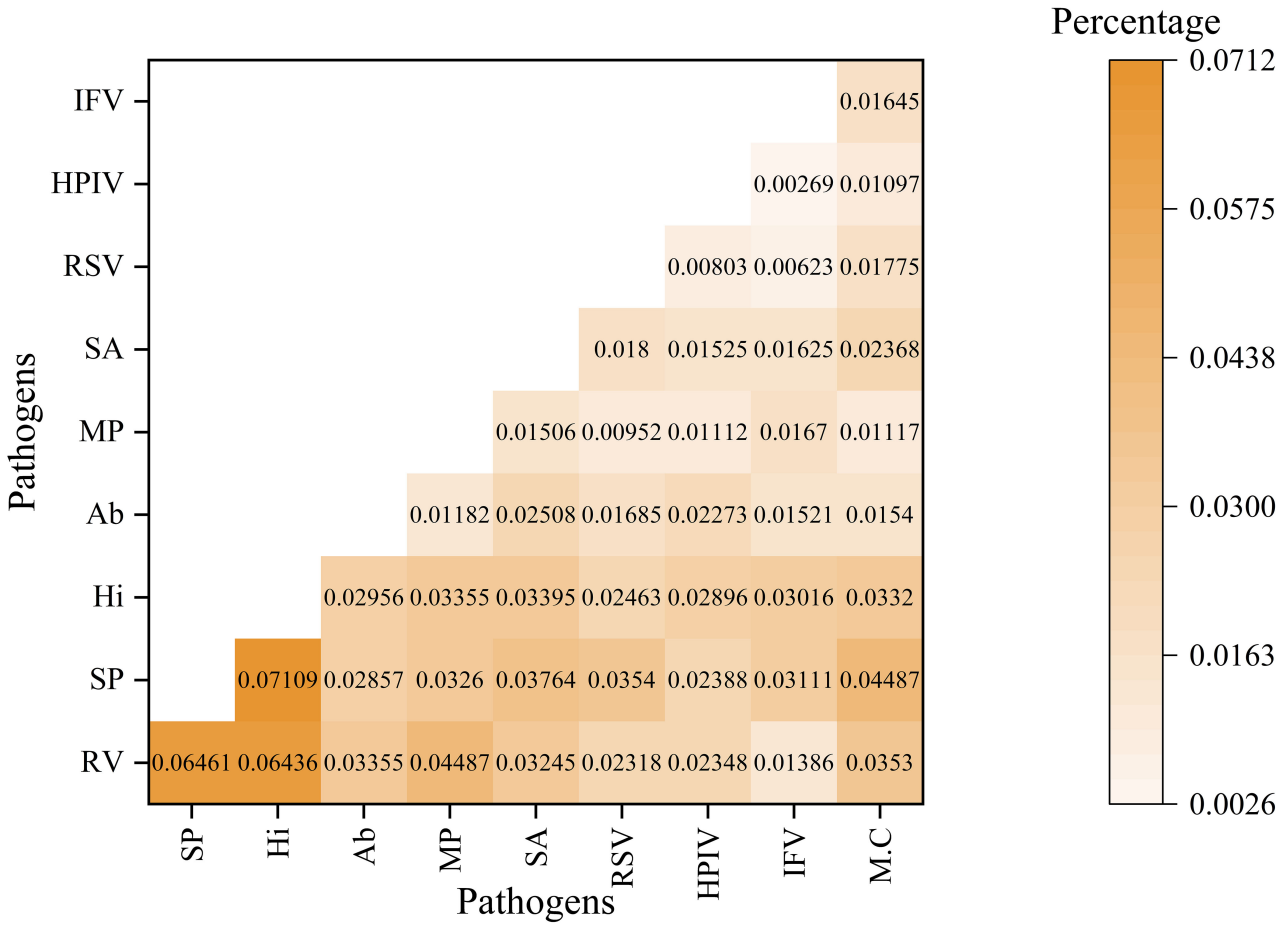
The co-detection rate analysis for top 10 pathogens This heatmap illustrates the co-detection percentages of various pathogen pairs. Both the rows and columns represent different pathogens. This color gradient reflects the proportion of samples in which both the row and column pathogens are detected simultaneously. The numerical value in each cell denotes the specific co-detection percentage for that pathogen pair.

The analysis of pathogen co-occurrence revealed complex interaction patterns (**Figure 5**). The predominant finding was bacterial-viral co-infection, which accounted for 49.03% of the cases, followed by bacterial infections alone at 15.69%. Notably, triple co-detection involving viruses, bacteria, and other pathogens including Mycoplasma pneumoniae, Chlamydia pneumoniae, Chlamydia psittaci, Chlamydia trachomatis, Ureaplasma urealyticum, Ureaplasma parvum, Mycoplasma hominis, Mycoplasma genitalium was observed in 5.77% of the cases, while single viral infections represented 13.82% of the detections. The co-detection analysis of the top ten pathogens demonstrates that common co-detection patterns frequently involve combinations of bacteria (e.g., Hi and SP) (**Figure 6**). The clinical significance of these co-infections, particularly their potential impact on disease severity, warrants further investigation.

### 3.7 Detection rates of respiratory pathogens across different specimen types

**Figure 7:**
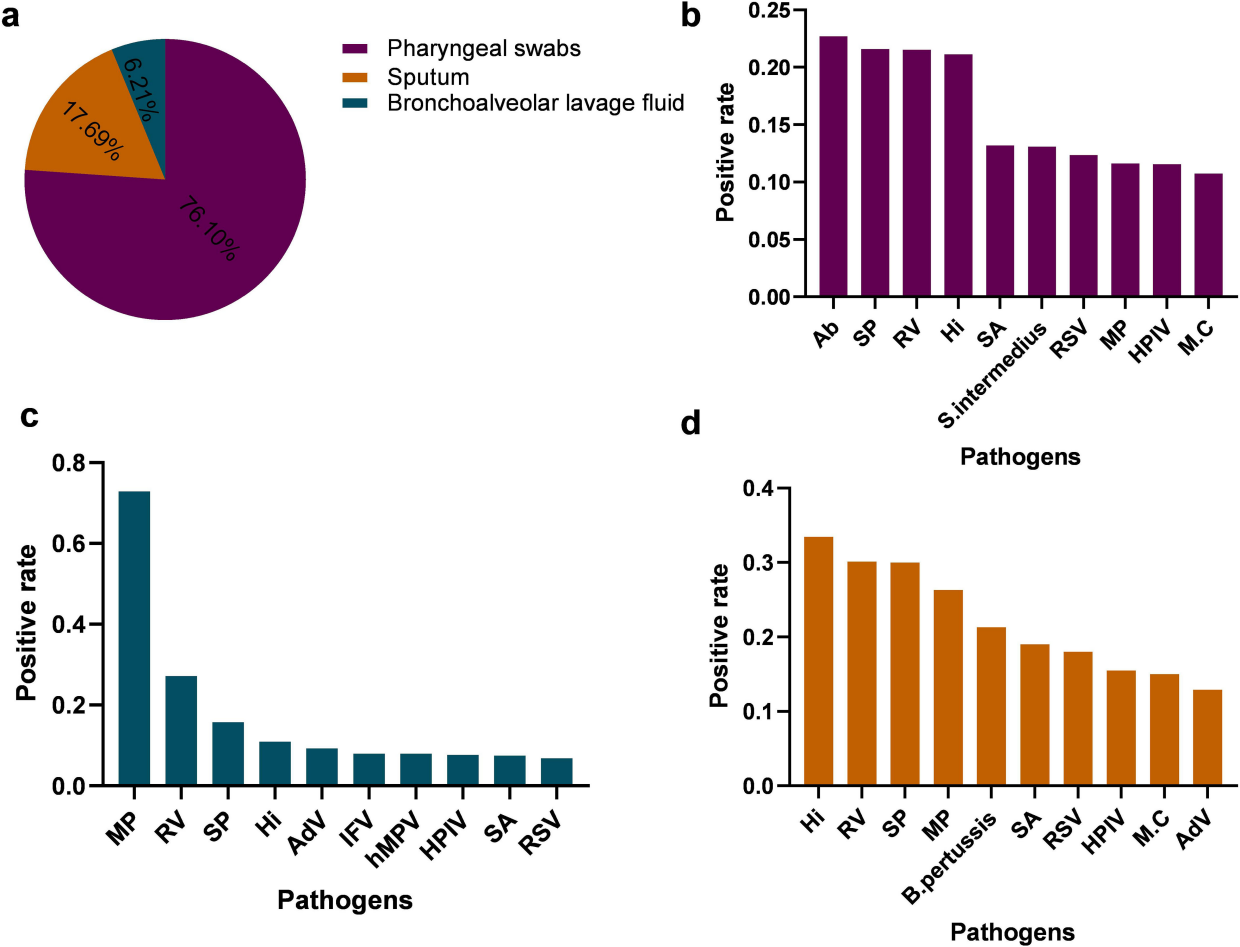
Comparison of positive detection rates of respiratory pathogens in different respiratory specimens **(a)** illustrate the types and distribution of respiratory specimens. **(b–d)** presents the positive rates and distribution of the top ten pathogens identified in pharyngeal swab specimens, Bronchoalveolar Lavage Fluid,Sputum, respectively. S. intermedius, Streptococcus intermedius; AdV, Adenovirus; hMPV, Human metapneumovirus; B. pertussis, Bordetella pertussis.

The positive detection rates of the ten most prevalent pathogens in each specimen type are summarized in **Figure 7**. Notably, the pathogen profile varied considerably across different specimen types. Acinetobacter baumannii (Ab) and Streptococcus pneumoniae (SP) were the most frequently detected in pharyngeal swabs (**Figure 7b**), whereas Haemophilus influenzae (Hi) and Rhinovirus(RV) predominated in sputum samples (**Figure 7d**). In bronchoalveolar lavage fluid, Mycoplasma pneumoniae (Mp) remained among the most commonly identified pathogens (**Figure 7c**).These findings underscore not only the differential detection rates of respiratory pathogens but also the substantial influence of specimen type on pathogen identification, highlighting the importance of appropriate sample selection in the diagnosis of respiratory infections.

## 4 Discussion

This study employs targeted Next-Generation Sequencing (tNGS) technology to provide a comprehensive overview of the respiratory pathogen landscape within the investigated cohort. It highlights significant associations with patient age, seasonality, and the prevalence of co-infections. The capability of tNGS to detect a wide array of pathogens presents substantial advantages over conventional diagnostic methods, which typically focus on a limited number of pathogens (Simões et al., 2013; Zumla et al., 2014; Li et al., 2021).

The spectrum of identified pathogens, including common viruses such as Rhinovirus, Influenza, and RSV, as well as bacteria like S. pneumoniae and other pathogens such as M. pneumoniae, aligns broadly with findings from other epidemiological studies conducted in China and globally (Li et al., 2021; Zhang et al., 2023; Li et al., 2025). However, the use of tNGS facilitates the detection of less common or unexpected pathogens, and the higher-resolution data provided by tNGS enhances the understanding of local infection characteristics.

The lack of a statistically significant overall difference in pathogen detection by gender (P = 0.2438 when comparing positive versus negative groups for overall pathogen presence) is noteworthy. However, males exhibited greater susceptibility to Rhinovirus, Streptococcus pneumoniae and Human parainfluenza virus. Previous studies (Chang et al., 2006; Xu et al., 2009) have indicated no significant difference in the prevalence of Mycoplasma pneumoniae infection between males and females; however, our study, along with another study (Yue et al., 2025), found that females were more susceptible to M. pneumoniae. Furthermore, it is crucial to examine gender-specific prevalence for individual pathogens, as hormonal, immunological, or behavioral differences may contribute to varying susceptibilities or exposure levels to specific agents (Oertelt-Prigione, 2012; Moulton, 2018; Sparks et al., 2023). Therefore, further investigation into the mechanisms of host-pathogen interactions is warranted.

Age-related findings are particularly striking. The high proportion of positive cases in the 0-1year age group (24.61%) and other young pediatric groups underscores their vulnerability to respiratory tract infections (RTIs). Specific pathogens, such as respiratory syncytial virus (RSV), are known to predominantly affect infants (Zhang et al., 2023; Tang et al., 2025), while agents like Mycoplasma pneumoniae are more common in older children and young adults (Li et al., 2025). Age-specific analyses revealed that Acinetobacter baumannii, human respiratory syncytial virus, and parainfluenza virus were more frequently detected in infants, while Streptococcus pneumoniae, influenza virus, and Moraxella catarrhalis were more prevalent in preschool-aged children.The elevated detection rate of Haemophilus influenzae among children aged 3–6 years may be linked to the sample type, as Haemophilus influenzae typically colonizes the upper respiratory tract of asymptomatic healthy children, making it detectable in pharyngeal swab samples. In contrast to other pathogens, Mycoplasma pneumoniae exhibited a higher detection rate in children aged 6–10 years, potentially linked to clustered transmission in school settings (Blasi, 2004; Li et al., 2025). These age-related findings likely reflect factors such as immune system maturity, waning maternal antibodies, and varying exposure patterns (Thomas, 2013).

The temporal distribution of pathogens aligns with the established seasonality observed in numerous respiratory agents. For instance, influenza virus (IFV) and respiratory syncytial virus (RSV) typically exhibit peak activity during the cooler months (Zhang et al., 2023; Li et al., 2025a), which may be attributed to the favorable conditions that lower temperatures create for viral transmission. The consistently high detection rates of Mycoplasma pneumoniae (MP), ranging from 38.6% to 60.7% between November 2023 and January 2024, may be linked to immune gaps or pathogen mutations. Understanding these local seasonal patterns is crucial for public health preparedness, particularly in the context of vaccination campaigns and resource allocation (Li et al., 2025b).

The frequent detection of co-infections represents a significant finding, underscoring the polymicrobial nature of many respiratory tract infections (RTIs). Co-detections involving viruses and bacteria are common and may modulate disease severity and clinical outcomes through mechanisms such as viral-induced mucosal barrier damage or host immune modulation (Lanjuan, 2023; Li et al., 2025). For instance, a primary viral infection can predispose an individual to secondary bacterial pneumonia. While targeted next-generation sequencing (tNGS) effectively identifies multiple pathogens, distinguishing true synergistic co-infections from bystander colonization or transient detection necessitates careful clinical correlation and potentially quantitative assessments of pathogen loads (Thomas, 2013).

The findings from this tNGS-based study hold significant implications for public health. These findings enhance our understanding of the burden of respiratory tract infections (RTIs), the circulation of pathogens, and the identification of high-risk populations. Such data can inform the development of targeted prevention strategies, including age-specific vaccination recommendations and the timing of public health advisories in relation to seasonal peaks. Additionally, monitoring co-infection patterns can inform empirical treatment strategies and bolster antimicrobial stewardship efforts.

The strengths of this study include the utilization of comprehensive targeted next-generation sequencing (tNGS) technology, the analysis of a substantial number of pathogen-positive samples (N = 19,474), and the examination of multiple epidemiological factors, including various specimen types. However, certain limitations must be acknowledged. As this study is a retrospective analysis of laboratory data, detailed clinical outcomes or severity scores for individual patients were likely unavailable for correlation with pathogen findings. The tNGS method detects nucleic acids, which does not always distinguish between active infections, colonization, or remnants of past infections. Furthermore, the “Negative group” exhibited a significantly different distribution in age and specimen types compared to the “Positive group,” suggesting that it may not represent a typical asymptomatic or healthy control population. Instead, it likely comprises individuals who were tested and found negative for the targeted pathogens; this aspect limits its utility for certain comparative epidemiological inferences without further characterization. Lastly, the findings may be specific to the geographical region and healthcare setting from which the samples were sourced, potentially restricting their generalizability without multi-center data.

Future research should focus on prospective studies that integrate targeted next-generation sequencing (tNGS) findings with comprehensive clinical data, including patient outcomes and host immune responses. Additionally, longitudinal surveillance utilizing tNGS can effectively monitor pathogen evolution and the emergence of novel threats.

## 5 Conclusion

tNGS offers a comprehensive methodology for identifying respiratory pathogens, thereby elucidating intricate epidemiological patterns. This study highlights significant correlations between pathogen profiles and variables such as patient age, seasonality, and the notable prevalence of co-infections. These findings are essential for comprehending local dynamics of respiratory tract infections (RTIs) and can guide targeted diagnostic, therapeutic, and public health strategies.

## Data Availability

The raw data supporting the conclusions of this article will be made available by the authors, without undue reservation.
