# Supplementary material for: tNGS-based detection of respiratory pathogens in a single center: associations with age, gender, season, and co-infections
Source: Front Cell Infect Microbiol. 2025 Nov 25;15:1663234. doi: 10.3389/fcimb.2025.1663234 (PMC12685647; doi:10.3389/fcimb.2025.1663234)
Supplement: Supplementary file 1 [file Table1.docx]

Supplementary Table S1 Spectrum of 107 targeted respiratory pathogens

| Virus | Bacteria | Atypical pathogens |
| --- | --- | --- |
| Human adenovirus | Cryptobacterium haemolyticum | Mycoplasma pneumoniae |
| Human adenovirus group A | Corynebacterium diphtheriae | Mycoplasma hominis |
| Human adenovirus group B | Corynebacterium ulcerans | Mycoplasma genitalium |
| Human adenovirus group C | Staphylococcus aureus | Chlamydia pneumoniae |
| Human adenovirus group D | Streptococcus agalactiae | Chlamydia psittaci |
| Human adenovirus group E | Streptococcus anginosus | Chlamydia trachomatis |
| Human adenovirus type 1 | Streptococcus dysgalactiae | Ureaplasma urealyticum |
| Human adenovirus type 2 | Streptococcus intermedius | Ureaplasma parvum |
| Human adenovirus 3 | Streptococcus pneumoniae |  |
| Human adenovirus 4 | Streptococcus pyogenes |  |
| Human adenovirus 5 | Listeria monocytogenes |  |
| Human adenovirus 7 | Acinetobacter baumannii |  |
| Human adenovirus 8 | Bordetella pertussis |  |
| Human adenovirus 12 | Fusobacterium necrophorum |  |
| Human adenovirus 18 | Haemophilus influenzae |  |
| Human adenovirus 21 | Haemophilus influenzae type B |  |
| Human adenovirus 24 | Klebsiella pneumoniae |  |
| Human adenovirus 27 | Legionella pneumophila |  |
| Human adenovirus 28 | Moraxella catarrhalis |  |
| Human adenovirus 30 | Neisseria gonorrhoeae |  |
| Human adenovirus 31 | Neisseria meningitidis |  |
| Human adenovirus 34 | Pseudomonas aeruginosa |  |
| Human adenovirus 38 | Serratia marcescens |  |
| Human adenovirus 55 | Stenotrophomonas maltophilia |  |
| Herpes simplex virus type 1 | Yersinia enterocolitica |  |
| Herpes simplex virus type 2 |  |  |
| Human herpesvirus 3 |  |  |
| Epstein-Barr virus |  |  |
| Human herpesvirus 5 |  |  |
| Human herpesvirus 6 |  |  |
| Human herpesvirus 6A |  |  |
| Human herpesvirus 6B |  |  |
| Human herpesvirus 7 |  |  |
| Human bocavirus type 1 |  |  |
| Human parvovirus B19 |  |  |
| Enterovirus |  |  |
| Enterovirus A |  |  |
| Enterovirus B |  |  |
| Enterovirus C |  |  |
| Enterovirus D |  |  |
| Enterovirus A71 |  |  |
| Enterovirus D68 |  |  |
| Coxsackievirus A5 |  |  |
| Coxsackievirus A6 |  |  |
| Coxsackie virus A10 |  |  |
| Coxsackie virus A16 |  |  |
| Echovirus 18 |  |  |
| Human coronavirus 229E |  |  |
| Human coronavirus HKU1 |  |  |
| Human coronavirus NL63 |  |  |
| Human coronavirus OC43 |  |  |
| Human metapneumovirus |  |  |
| Human respiratory syncytial virus A |  |  |
| Human respiratory syncytial virus B |  |  |
| Human parainfluenza virus 1 |  |  |
| Human parainfluenza virus 2 |  |  |
| Human parainfluenza virus 3 |  |  |
| Human parainfluenzavirus 4 |  |  |
| Influenza A virus |  |  |
| Influenza A virus H1N1 |  |  |
| Influenza A virus H3N2 |  |  |
| Influenza A virus H5N1 |  |  |
| Influenza A virus H7N9 |  |  |
| Influenza A virus H1N12009 |  |  |
| Influenza B virus |  |  |
| Influenza C virus |  |  |
| Measles virus |  |  |
| Mumps virus |  |  |
| Rhinovirus |  |  |
| Rhinovirus A |  |  |
| Rhinovirus B |  |  |
| Rhinovirus C |  |  |
| Rubella virus |  |  |
| SARS-Cov-2 |  |  |

Mycoplasma pneumoniae macrolide resistance gene locus: 23SrRNA: A2063G, 23SrRNA: A2064G, 23SrRNA:A2067G, 23SrRNA:C2617G.

Bordetella pertussis resistance gene / locus: 23S rRNA:A2047G.
